# In Vitro Anti-Inflammatory Activity of *Cotula* *anthemoides* Essential Oil and In Silico Molecular Docking of Its Bioactives

**DOI:** 10.3390/molecules27061994

**Published:** 2022-03-19

**Authors:** Mohamed S. Refaey, Mohamed E. Abouelela, Ehab A. M. El-Shoura, Hala M. Alkhalidi, Sana A. Fadil, Sameh S. Elhady, Reda F. A. Abdelhameed

**Affiliations:** 1 Department of Pharmacognosy, Faculty of Pharmacy, University of Sadat City, Sadat City 32897, Egypt; 2 Department of Pharmacognosy, Faculty of Pharmacy, Al-Azhar University, Assiut Branch, Assiut 71524, Egypt; m_abouelela@azhar.edu.eg; 3 Department of Pharmacology & Toxicology, Faculty of Pharmacy, Al-Azhar University, Assiut Branch, Assiut 71524, Egypt; ehabel-shoura@azhar.edu.eg; 4 Department of Pharmacy Practice, Faculty of Pharmacy, King Abdulaziz University, Jeddah 21589, Saudi Arabia; halkhaldi@kau.edu.sa; 5 Department of Natural Products, Faculty of Pharmacy, King Abdulaziz University, Jeddah 21589, Saudi Arabia; safadil@kau.edu.sa; 6 Department of Pharmacognosy, Faculty of Pharmacy, Galala University, New Galala 43713, Egypt; reda.fouad@gu.edu.eg; 7 Department of Pharmacognosy, Faculty of Pharmacy, Suez Canal University, Ismailia 41522, Egypt

**Keywords:** Asteraceae, phytoconstituents, tumor necrosis factor, cyclooxygenase, molecular modeling

## Abstract

The genus *Cotula* (Asteraceae) comprises about 80 species, amongst them *Cotula anthemoides* L. It is a wild plant growing in Egypt that possesses many traditional uses as a headache, colic, and chest cold remedy. In our study, the chemical composition of *C*. *anthemoides* essential oils was analyzed using GC-MS spectroscopy. Sixteen components of leave and stem oils and thirteen components of flower oils were characterized. The main components in both essential oil parts were camphor (88.79% and 86.45%) and *trans*-thujone (5.14% and 10.40%) in the leaves and stems and the flowers, respectively. The anti-inflammatory activity of the oils in lipopolysaccharide-stimulated RAW 264.7 macrophage cells was evaluated. The flower oil showed its predominant effect in the amelioration of proinflammatory cytokines and tumor necrosis factor-α, as well as cyclooxygenase-2. The bornyl acetate showed the highest affinity for the cyclooxygenase-2 receptor, while compound *cis*-*p*-menth-2-ene-1-ol had the best affinity for the tumor necrosis factor receptor, according to the results of molecular docking. In addition, the molecule *cis*-*β*-farnesene showed promising dual affinity for both studied receptors. Our findings show that essential oils from *C*. *anthemoides* have anti-inflammatory properties through their control over the generation of inflammatory mediators. These findings suggest that *C*. *anthemoides* essential oils could lead to the discovery of novel sources of anti-inflammatory treatments.

## 1. Introduction

Inflammation is a healthy immune response that, under normal circumstances, defends against a variety of diseases and traumas by minimizing tissue injury [1]. However, chronic inflammation leads to the dysregulation of the immune cells, which is associated with various chronic diseases such as cancer, rheumatoid arthritis, osteoarthritis, ulcerative colitis, dermatitis, inflammatory bowel disease, Alzheimer’s, systemic lupus erythematous, and cardiovascular diseases [2,3]. Immune cells, particularly macrophages, play a crucial role in the response to external stimuli during inflammation. In macrophages, lipopolysaccharide (LPS), an endotoxin, induces the secretion of inflammatory cytokines such as tumor necrosis factor alpha (TNF-*α*), interleukin-1 (IL-1), and interleukin-6 (IL-6) [1]. Inflammatory mediators such as inducible nitric oxide (iNO), prostaglandin E2, and cyclooxygenase-2 (COX-2) are also implicated in the onset and advancement of the inflammatory process [2,4]. As a result, the level of pro-inflammatory mediators and cytokines is connected to the intensity of inflammation and is used to evaluate the influence of medications on inflammatory processes [5]. Anti-inflammatory and antioxidant agents are thought to be a practical mechanism for reducing the impact of chronic inflammation in the progression of degenerative diseases [6]. These challenges require the development of innovative medicines that are more effective and less hazardous in the treatment of acute and chronic inflammatory disorders [7]. As a consequence of the negative side effects of non-steroidal anti-inflammatory drugs (NSAIDs), plant extracts and essential oils are being investigated as a potential new anti-inflammatory drug scaffold that can inhibit the release of inflammatory mediators and free radicals and boost antioxidant defenses [8,9]. With about 1600 genera and 22,000 species, the Asteraceae is the biggest flowering plant family [10]. It has numerous cost-effective applications in foods, cosmetics, and pharmaceuticals. Asteraceae plants have been shown to have anti-inflammatory properties in several investigations [10].

**Figure 1:**
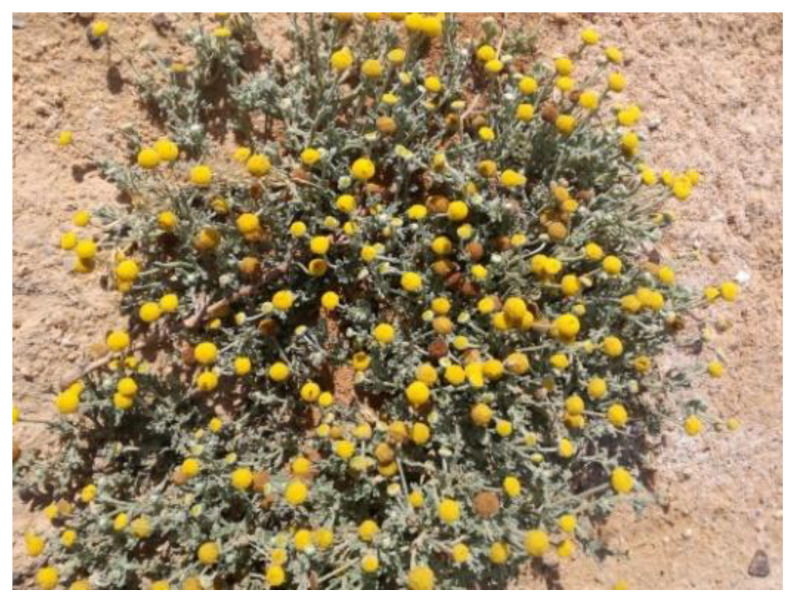
Photograph of *C. anthemoides* taken at the time of collection.

The genus *Cotula* is of the Asteraceae family and consists of about 80 species, mostly endemic to South Africa [11]. *Cotula* species are traditionally used as anti-inflammatory, antipyretic, antiprotozoal, analgesic, bacteriostatic, or antiseptic agents and in the treatment of digestive disorders [12,13,14,15]. *Cotula anthemoides* L. (Figure 1), is used in traditional medicine as a headache, colic, and chest cold remedy [16]. The leaf decoction used in combination with *Prunella vulgaris* (Lamiaceae) and *Salix alba* (Salicaceae) cures rheumatism and body pain [17]. Different extracts of the roots and aerial parts were found to be effective against *Staphylococcus aureus* [18]. Furthermore, people living in remote areas used the plant’s hot water paste to treat inflammation caused by fractures. It is also used for pulmonary and stomach troubles [19]. Despite the various traditional uses of this plant, there is a lack of biological investigations, with only a few phytochemical studies concerning it. This encourages us to assess its biological activity as a potential new source for the treatment of inflammation and inflammation-related diseases.

The goal of this work was to look into the cytoprotective and anti-inflammatory properties of *C. anthemoides* essential oil CAEO on LPS-induced RAW 264.7 macrophage cell cultures. These findings will, to the best of our knowledge, show for the first time the putative anti-inflammatory mechanism provided by CAEO.

## 2. Results and Discussion

### 2.1. Essential Oil Composition

**Figure 2:**
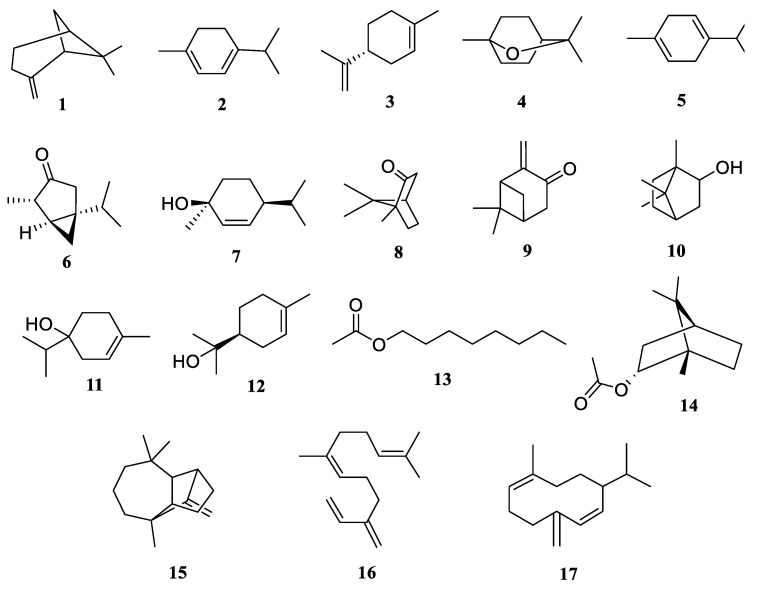
Chemical structure of identified compounds in *C. anthemoides* essential oil. (**1**, *β*-Pinene; **2**, *α*-Terpinene; **3**, D-Limonene; **4**, Eucalyptol; **5**, γ-Terpinene; **6**, *trans*-Thujone; **7**, *cis*-*para*-Menth-2-ene-1-ol; **8**, Camphor; **9**, Pinocarvone; **10**, Borneol; **11**, Terpinen-4-ol; **12**, *α*-Terpineol; **13**, Octanol acetate; **14**, Bornyl acetate; **15**, Longifolene; **16**, *cis*-*β*-Farnesene; **17**, Germacrene D).

The hydrodistillation of CAEO led to the recovery of yellowish-colored essential oil residues with a fragrant odor yielding 0.18 % *v*/*w* for *C. anthemoides* leaves and stem essential oils (CALSO), and 0.27% *v*/*w* for *C. anthemoides* flower essential oils (CAFO). The analysis of the obtained essential oil’s composition (Table 1, Figure 2) was conducted by gas liquid chromatography mass spectroscopy (GLC-MS). The oxygenated monoterpenes predominated in the constituents of CAEO, accounting for 96.72% and 97.87% for CALSO and CAFO, respectively. The major oxygenated monoterpenes were camphor (88.79%, CALSO and 86.45%, CAFO) and thujone (5.14%, CALSO and 10.40%, CAFO), while the oxygenated monoterpene, borneol, (0.11%) was only detected in the flower essential oil and was absent in the leave and stem parts. On the other hand, the sesquiterpene compounds longifolene, *cis*-*β*-Farnesene, and Germacrene D, as well as, the monoterpene compound *α*-Terpineol, were detected in the leaves and stem essential oil components and absent in the flower essential oil. The other detected constituents are shown in (Table 1, Figure 2).

A previous study on essential oil obtained by hydrodistillation from the aerial parts of Algerian *C. anthemoides* found it to be rich in monoterpenes. They reported that the major oil components of the aerial parts were camphor (27.4%) and thujone (12.9%), in addition to santolinatriene (13.0%), camphene (10.7%), and curcumene (5.3%) [20]. Furthermore, our findings contrast with the previously published essential oil composition of *Cotula coronopifolia* L. leaves, which was dominated by agarospirol (10.4%), while hexacosane (31.7%) and 1-eicosanol (17.1%) were prevalent in the flower and stem, respectively. Heptacosane (28.4%), 1-eicosanol (14.6%), octacosane (5.4%), and γ-amorphen (5.2%) were the primary compounds found in the root oil [21].

The geographic location, climatic impacts, harvest season, nature of the soil, age of the plant parts (young or mature), state of the used plant materials (dry or fresh), part of the plant used, time of collection, and chemotype are all elements that might affect the chemical makeup of essential oils [22,23,24].

### 2.2. Cytotoxicity of C. anthemoides Essential Oils against RAW 264.7 Cells

**Figure 3:**
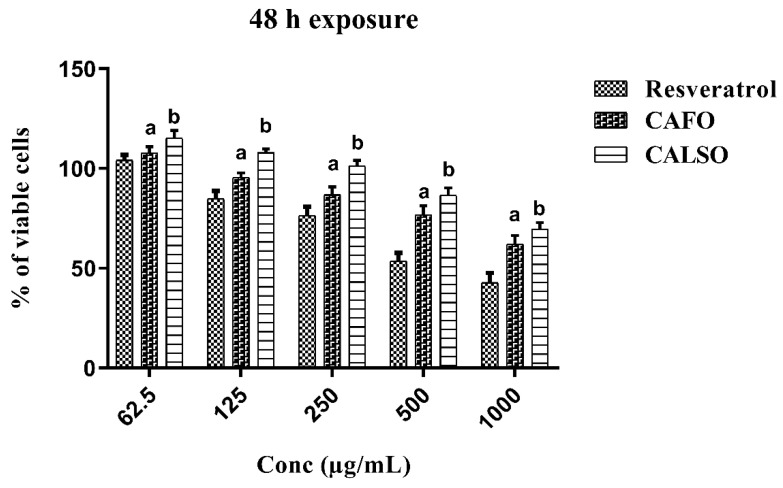
The effects of *C. anthemoides* essential oils on the cytotoxicity of RAW 264.7 cells in comparison to resveratrol in different concentrations for 48 h (*n* = 3). Each bar represents the mean ± S.E.M. Multiple comparisons were completed using two-way ANOVA, followed by Tukey–Kramer as the post-ANOVA test. ^a^ Significantly different from the value of the resveratrol group at *p* < 0.05. ^b^ Significantly different from the value of the CAFO group at *p* < 0.05. CAFO: *C. anthemoides* flower essential oil; CALSO: *C. anthemoides* leaves and stem essential oil.

The CAEO’s toxicity against RAW 264.7 cells was evaluated by a 3-(4,5-dimethylthiazol-2-yl)-2,5-diphenyl-2H-tetrazolium bromide (MTT) assay. RAW 264.7 cells were treated with various concentrations (1000, 500, 250, 125 and 62.5 µg/mL) of CALSO and CAFO for 48 h. As shown in Figure 3, a slight toxicity was observed in a dose-dependent manner, but the RAW 264.7 cells’ viability was not significantly decreased, with levels up to 250 µg/mL. The CALSO showed the lowest toxic effect, followed by CAFO, with IC_50_ values of 1499.8 ± 82.8 and 2627.30 ± 14.5 µg/mL, respectively, in comparison to resveratrol (IC_50_ = 914.38 ± 50.5 µg/mL). Therefore, CALSO and CAFO are not significantly toxic to RAW 264.7 cells. As a result of these findings, it is plausible to conclude that CAEO has a favorable safety profile, justifying and encouraging its use as condiment, food preservative, and aliments remedy.

### 2.3. Effects of C. anthemoides Essential Oils on COX-2 Levels against LPS-Stimulated RAW 264.7 Cells

**Figure 4:**
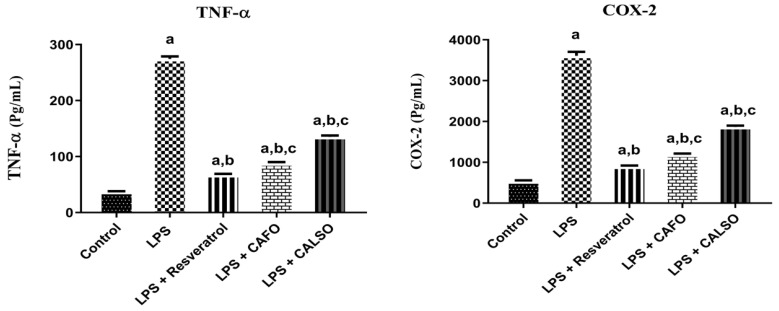
Effect of essential oils on COX-2 and TNF-α levels in LPS-induced inflammation in RAW264.7 cells. Data are expressed as means ± SEM (*n* = 3), and analyzed by using one-way ANOVA followed by Tukey–Kramer as the post-ANOVA test. ^a^ Significantly different from the value of the control group at *p* < 0.05. ^b^ Significantly different from the value of the LPS group at *p* < 0.05. ^c^ Significantly different from the value of the LPS + resveratrol group at *p* < 0.05. LPS: Lipopolysaccharides; CAF: *C. anthemoides* flower essential oil; CALSO: *C. anthemoides* leaves and stem essential oil.

To establish the effects of CAEO on LPS-induced COX-2 production, changes in COX-2 activity in response to LPS-stimulated RAW 264.7 cells treated with essential oils from different parts (10 µg/mL) were evaluated (Figure 4). The inflammation induced by the addition of LPS markedly elevated the activity of COX-2 by 573.57% with respect to the normal control group. Cell lysate treated with resveratrol showed significantly suppressed levels of COX-2, down by 76.17% in comparison to LPS-treated RAW 264.7 cells. On the other hand, CAEO treatment of either the flower or the leaves and stem showed noticeably suppressed COX-2 activities, down by about 67.03% and 48.32%, respectively, in comparison with the LPS-treated group. The results indicate that CAEO may suppress the inflammatory response by mitigating the activity of COX-2 in LPS-stimulated RAW 264.7 cells.

### 2.4. Effects of C. anthemoides Essential Oils on TNF-α Levels in LPS-Stimulated RAW 264.7 Cells

To assess the effect of CAEO on proinflammatory cytokine TNF-*α* levels, we investigated their effects on TNF-*α* in LPS-activated RAW 264.7 cells. Recent data have demonstrated that LPS-induced inflammation is strongly linked to a variety of intracellular signaling pathways, such as nuclear factor kappa B, which regulates a number of inflammatory genes including TNF-α. The cells were stimulated by LPS in the presence of the essential oil of different parts The TNF-*α* concentration in the supernatants was measured using the ELISA technique. As shown in Figure 4, LPS-treated cells showed significantly elevated levels of TNF-*α*, up by about 639.22% in comparison to the normal control group. Cells treated with resveratrol significantly reversed the level of TNF-α, down by 75.50% compared to LPS-treated RAW 264.7 cells. Treatment with 10 μg/mL of *C. anthemoides* essential oil on either the flower or the leaves and stem significantly attenuated the levels of TNF-*α* by 67.86% and 50.72%, respectively, with respect to the LPS-treated cells.

According to the findings related to the anti-inflammatory effects of CAEO on COX-2 and TNF-α levels in LPS-stimulated RAW 264.7 cells, we can attribute this activity to its major oxygenated monoterpene constituents: camphor and thujone. This can be obvious from previous studies regarding the anti-inflammatory effects of different plants’ essential oils, where the major active components were also camphor and thujone. A study reported by Yoon et al. (2010), [25] showed that camphor and alpha- and beta-thujone, the major components of *Artemisia fukudo* (Asteraceae) essential oil, could inhibit the release of TNF-α, IL-1 β, and IL-6 in LPS-treated RAW 264.7 cells. Homnan et al. demonstrated that D(+)-camphor, the major constituent of lesser galangal essential oil, significantly inhibited COX-2 activity at different rates of up to 78.34% in LPS-induced inflammation in PMA-differentiated THP-1 macrophages [26]. The most abundant components in *Salvia officinalis* (Lamiaceae) essential oils gathered from northern and southern Albania were camphor, *α*-thujone, and 1,8-cineole. In RAW 264.7 cells, the anti-inflammatory evaluation of essential oils extracted from both sources revealed that both oils dramatically reduced nitric oxide (NO) and nuclear kappa B (NF-κB) production [27]. Furthermore, studies of the anti-inflammatory effect of *Artemisia sieberi* (Asteraceae) oil in a paw edema experiment on rats revealed that a 1-mg/kg dose of the oil significantly reduced carrageenan-induced paw edema in rats in the first hour of the test by 72.7%, which lasted until the third hour of the test [28].

In the previous study, hydroethanolic and infusion extracts of *Cotula cinerea* (Asteraceae) reduced nitric oxide production in Murine macrophage-like RAW 264.7 cells with EC_50_ values of 105 ± 9 and 122 ± 6 µg/mL, respectively [29]. These findings could be linked to the genus’ widespread use in traditional medicine [18,19].

### 2.5. In Silico Molecular Docking Simulation

In our study, seventeen GLC-MS-identified compounds (Table 1) were virtually screened against human cyclooxygenase-2 (COX-2) (PDB ID: 5KIR) and the tumor necrosing factor (TNF-*α*) (PDB ID: 2AZ5) using a molecular docking simulation, to discover their possible anti-inflammatory activity through inhibitions of selected inflammatory mediator proteins. The interactions of these substances with the active site residues of these proteins were investigated. The poses of the docked ligand conformers were filtered by the root-mean-square deviation (RMSD) to determine their experimental stability [30], and RMSD values around 1.5 Å were considered significant [31]. The results are illustrated in (Table 2).

**Figure 5:**
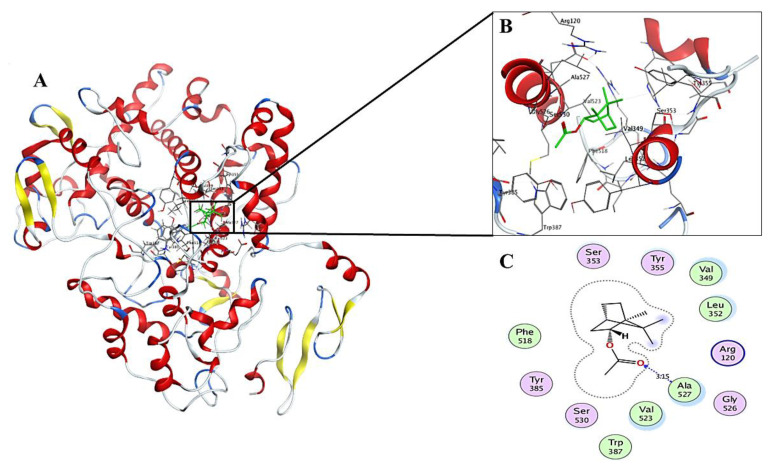
2D and 3D interactions complex of compound **14** (bornyl acetate) with the COX-2 receptor. (**A**) receptor ligand complex, (**B**) 3D interactions of ligand with the active site, (**C**) 2D interactions of ligand with the active site.

The analysis of the results revealed that the poses of the interactions between the compounds and the COX-2 receptor showed moderate-to-high affinities ranging from −9.6206 to −7.2938 kcal/mol, in comparison to resveratrol as a standard inhibitor (−11.2915 kcal/mol). The compound bornyl acetate showed the highest affinity for the receptor with a pose score of −9.6206 (RSMD = 0.91). The involved interactions depicted in Figure 5, which demonstrates the formation of a hydrogen bond between the carbonyl group and the Ala 527 amino acid residue, reduced the interaction energy score and stabilized the complex. The hydrophobic interactions with the Val 349, Leu352, Phe 518, Val 523, and Trp 387 amino acid residues are also included. In vivo and in vitro anti-inflammatory studies of bornyl acetate exhibited its ability to suppress ear swelling caused by dimethylbenzene and the induction of IL-11 in human chondrocytes, respectively [28,32].

**Figure 6:**
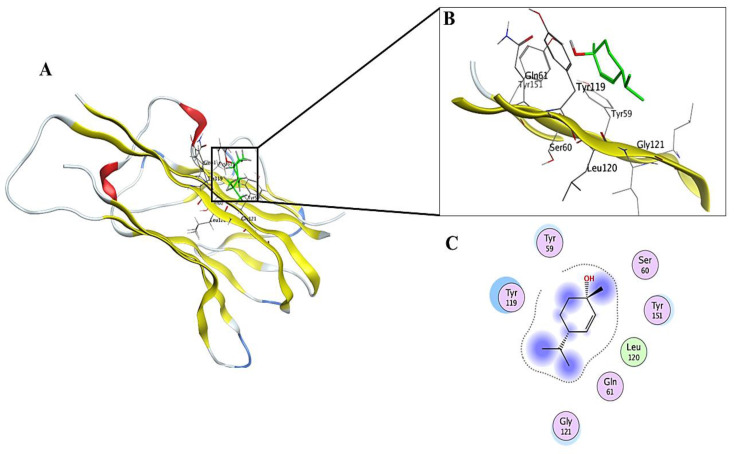
2D and 3D interactions complex of compound **7** (*cis*-*para*-Menth-2-ene-1-ol) with the TNF-*α* receptor. (**A**) receptor ligand complex, (**B**) 3D interactions of ligand with the active site, (**C**) 2D interactions of ligand with the active site.

Regarding the TNF-*α* receptor, the pose interactions (Table 2) showed that the pose core ranged from −6.9306 kcal/mol for the standard compound resveratrol to −4.4942 kcal/mol for *β*-Pinene. The compound *cis*-*para*-Menth-2-ene-1-ol has the most promising affinity, with a pose score of −6.7740 kcal/mol (RSMD = 1.25) in comparison to resveratrol (−6.9306 kcal/mol). The compound’s interactions with the receptor are shown in (Figure 6).

**Figure 7:**
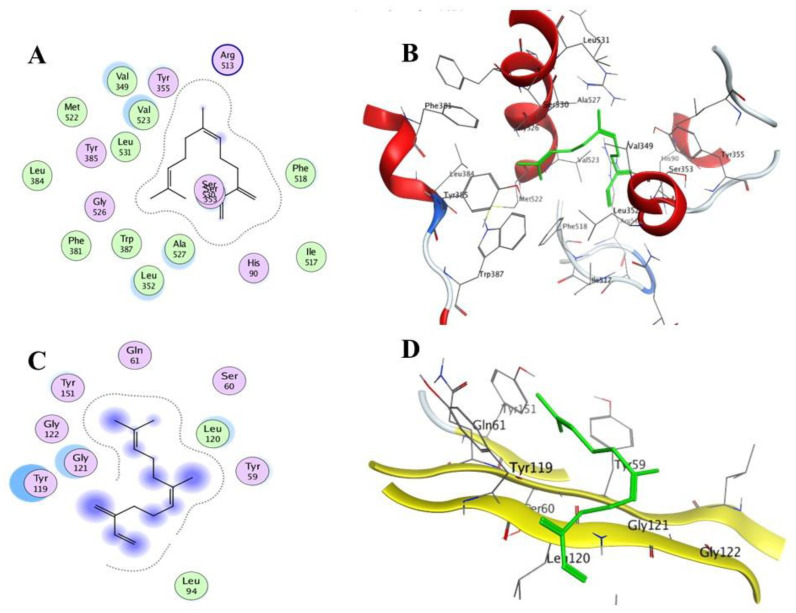
2D and 3D interactions complex of compound **16** (*cis*-*β*-Farnesene) with the COX-2 (**A**,**B**) and TNF-*α* (**C**,**D**) receptors’ active sites.

It is also worth noting that the compound *cis*-*β*-Farnesene demonstrated promising dual affinity with the tested receptor, with pose scores of −9.4392 kcal/mol (RSMD = 1.09) for COX-2 and −5.9222 kcal/mol (RSMD = 1.35) for TNF-*α* (Figure 7). It could be considered as a promising scaffold for developing new anti-inflammatory drug candidates.

### 2.6. Structure Drug-like Properties

The fast method for assessing the drug-like properties of a bioactive compound is to apply Lipinski’s rule [33]. According to the rule, “most drug-like compounds have logP = 5, number of hydrogen bond acceptors ≤10, and number of hydrogen bond donors ≤5, molecular weight ≤500, and molecules that violate more than one of these principles may have bioavailability difficulties” [33]. By inspecting the anticipated rule of having five findings for the tested compounds, all compounds were compiled according to Lipinski’s criteria except for longifolene, *cis*-*β*-Farnesene, and Germacrene D, which broke only one rule when their Log P exceeded the normal limit of 5 (Table 3).

## 3. Materials and Methods

### 3.1. General

COX-2 ELISA Kit was purchased from (BioVision, Milpitas Blvd., Milpitas, CA, USA) and TNF-α ELISA Kit was obtained from (MyBiosource, San Diego, CA, USA). In addition, LPS was also bought from Sigma-Aldrich (St. Louis, MO, USA).

### 3.2. Plant Material

*C. anthemoides* fresh leaves, stems, and flowers were collected from Sadat City, Menoufia, Egypt in February 2021. The plant material was recognized with the help of Dr. Mohamed F. Azzazy, professor of plant ecology at the Surveys of Natural Resources Department, Environmental Studies and Research Institute, University of Sadat City. A voucher specimen (Ca-En-USC 10) was placed in the Herbarium of Environmental Studies and Research Institute, Sadat City, Egypt.

### 3.3. Extraction of Essential Oil

The fresh leaves and stems (170 g) and flowers (260 g) of *C. anthemoides* were grounded in a blender (Braun, Kronberg im Taunus, Germany) and then subjected to hydro-distillation in a Clevenger-type apparatus under atmospheric pressure at a boiling temperature of about 100 °C for 3 h [34], and the distilled essential oils were collected and dried over anhydrous sodium sulfate (Lanxess, Thane, India). The oil samples were kept frozen (4 °C) in dark glass vials until further analysis [35].

### 3.4. Gas Liquid Chromatography-Mass Spectrometry (GLC/MS)

The composition of the essential oils was determined using a GLC-MS system (Agilent Technologies, Middelburg, The Netherlands) outfitted with a gas chromatograph (7890B), mass spectrometer detector (5977A), and HP-5MS column (30 m × 0.25 mm internal diameter and 0.25 μm film thickness) at Central Laboratories Network, National Research Centre, Cairo, Egypt. The temperature program was set to 40 °C for 1 min; then raised at 4 °C/min to 150 °C for 6 min; and lastly raised at 4 °C/min to 210 °C and held for 1 min. The injector and detector were both kept at 280 °C and 220 °C, respectively. Electron ionization (EI) at 70 eV was used to obtain the mass spectra with a spectral range of *m*/*z* 50–550 and a solvent delay of 5 min. The samples were diluted with *n*-hexane (≥99%, Sigma–Aldrich, Sternheim, Germany, 1:19, *v*/*v*) injected at 1 µL volume, and analyses were carried out using hydrogen as the carrier gas at a flow rate of 1.0 mL/min. The retention indices were calculated based on the retention times of the standard alkane series (C7–C40, Sigma Aldrich GmbH, Sternheim, Germany). The recording and integrating of the chromatograms was performed using enhanced Chem Station software, version MSD F.01.01.2317 (Agilent Technologies, Middelburg, The Netherlands). Based on the normalization method using the reading of three chromatographic runs, quantitation was carried out. The identification of the compounds was elucidated by comparing their retention indices (RIs) and mass spectral data with the reported data in the Wiley Registry of Mass Spectral Data (9th Ed.), NIST Mass Spectral Library (2011).

### 3.5. Cell Culture, Treatments, and MTT Assay

The RAW 264.7 macrophage cell line was obtained from the confirmatory diagnostic unit (VACSERA, Cairo, Egypt) and cultured in Dulbecco’s Modified Eagle’s Medium, which was purchased from (Invitrogen/Life Technologies, Carlsbad, CA, USA), and then supplemented with 10% fetal bovine serum, 100 U/mL penicillin, 100 μg/mL streptomycin, and 2 mML-glutamine (Thermo Fisher scientific, Carlsbad, CA, USA) at 37 °C in a 5% CO_2_ humidified air environment (Sigma-Aldrich; St. Louis, MO, USA). Finally, cell lysis was made by repeated freezing and thawing cycles. After one hour of pretreatment with and without various concentrations of CAEO, the cell lysate was treated with LPS (100 ng/mL) for the duration indicated. Cell viability was assessed by the MTT assay. Briefly, the cell lysate of RAW 264.7 cells (5 × 10^5^ cells/mL) was treated with the indicated concentrations of LPS (100 ng/mL) by itself, or pretreated with different concentrations of CAEO (1000, 500, 250, 125, and 62.5 µg/mL) for 1 h before LPS treatment. The media was withdrawn after 24 h, and cell lysis occurred after several freezing and thawing cycles. The cell lysate was incubated with 0.5 mg/mL MTT solution for 2 h, as described in the manufacturer’s protocol (in vitro toxicology assay kit MTT; Sigma-Aldrich, St. Louis, MO, USA) [36]. The supernatant was then discarded, and dimethyl sulfoxide (Fisher Scientific Company, Loughborough, UK) was used to dissolve the formazan blue that had developed. A microplate reader (ROBONIK P2000, Thane, India) was used to detect optical density at 540 nm. The essential oil’s cytotoxic activity was determined to reduce cell viability by 50% (IC_50_ value).

### 3.6. Assesment of Proinflammatory Biomarkers in LPS-Induced RAW 264.7 Macrophages

Proinflammatory mediators such as TNF-α level and COX-2 activity were estimated in the cell lysate by using commercially available enzyme-linked immunosorbent assay (ELISA) kits according to the manufacturer’s instructions [37,38]. RAW 264.7 macrophages (5 × 10^5^ cells/mL) were treated for 30 min with different concentrations of the essential oil samples (10 µg/mL), followed by incubation with or without 1 μg/mL LPS for 24 h. The optical density was measured at wavelength 450 nm by the ELISA assay (ROBONIK P2000, Thane, India).

### 3.7. In Silico Molecular Docking Simulation

In order to explore the binding and interaction modes of the compounds identified in volatile oil samples from both the leaves and stem and the flower for the selected inflammatory mediator proteins, molecular docking was performed using Molecular Operating Environment software ( v. 2014.09.01). The compounds were imported to MOE 2014 and subjected to 3D protonation and Merck molecular force field (MMFF94s) energy minimization, and were partially charged. A stochastic conformational search was conducted, the minimum dE conformers were selected, and a virtual ligand database was constructed. The crystal structures of the COX-2 (PDB ID: 5KIR; resolution 2.7 Å) [31] and TNF-α (PDB ID: 2AZ5; resolution 2.1 Å) [39] enzymes were obtained as pdb files from the Protein Data Bank (https://www.rcsb.org/, accessed on 26 June 2021). All the hetero atoms and unbound water molecules were removed from the target proteins, and their structures were optimized for docking simulation by the Quickprep built-in function.

The structures of the GLC-MS identified compounds (Table 1) were retrieved from NIST Chemistry WebBook SRD 69 (NIST Chemistry WebBook) as mol files. The obtained molecules were protonated and their energy minimized, and a combined database was generated to search for the best optimal binding against the selected targets.

The molecular docking simulations were conducted using optimized ligands and receptors according to the rigid receptor and flexible ligand MOE protocolThe parameters were set as Triangle Matcher for the placement function and London dG for both the scoring and rescoring functions. The docking poses were set at 30 poses for the initial energy score and 10 for refinement. For the interaction analysis, the docking score, root-mean-square deviation (RSMD), and ligand-receptor complexes were examined, and 2D and 3D images were obtained by the MOE visualizing tool [40].

### 3.8. Structure Drug-like Properties

Swiss ADME and PreADMET estimation websites were used to assess the drug likeness of the compounds and predict their molecular characteristics [41].

### 3.9. Statistical Analysis

Means and standard errors (S.E.M.) were used to express the data. To compare for significant changes between groups, one- and two-way ANOVA tests were used, followed by Tukey–Kramer multiple comparison tests across the five groups. For all comparisons, a significant level of *p* < 0.05 was used. All statistical testing was performed with the GraphPad Prism^®^ software program, version 7 (GraphPad software Inc., San Diego, CA, USA).

## 4. Conclusions

Essential oil derived from different parts of *C. anthemoides* (CAEO) revealed that the presence of the major components camphor and *trans*-thujone suppress the inflammatory response in LPS-stimulated RAW 264.7 cells by improving the COX-2 and lowering the TNF-*α* levels. According to experimental studies, the essential oil extracted from the flower parts has more anti-inflammatory effect than the sum of the essential oils extracted from the leaves and stems. The molecular docking results attributed this activity to the presence of bornyl acetate and *cis*-*para*-Menth-2-ene-1-ol, as well as *cis*-*β*-Farnesene, which could be used as lead compounds for the discovery of a selective anti-inflammatory drug. As a natural anti-inflammatory, several dosage forms of CAEO can be employed in pharmaceutical goods. Future phytochemical and pharmacological research on other *Cotula* species should expand on the findings of this study. This has the potential to be extremely important and pave the way for new therapeutic items. However, more research is needed to assess the usefulness of these species in industrial applications.

## Figures and Tables

**Table 1:** Chemical composition of *C. anthemoides* essential oils.

No	R_t_, min	Compound Name	Retention Index		Composition (%) **	
			(Cal.)	(Rep.) *	Leaves and Stems	Flowers
1	6.6	*β*-Pinene	976	974	0.31 ± 0.01	1.02 ± 0.12
2	7.9	*α*-Terpinene	1014	1014	0.26 ± 0.02	0.10 ± 0.01
3	8.3	D-Limonene	1026	1024	0.15 ± 0.09	0.26 ± 0.09
4	8.4	Eucalyptol	1032	1031	1.14 ± 0.06	0.42 ± 0.07
5	9.3	γ-Terpinene	1060	1059	0.52 ± 0.23	0.16 ± 0.03
6	9.8	*trans*-Thujone	1114	1112	5.14 ± 0.36	10.40 ± 0.57
7	11.4	*cis*-*para*-Menth-2-ene-1-ol	1120	1118	0.34 ± 0.03	0.17 ± 0.01
8	12.5	Camphor	1145	1141	88.79 ± 1.17	86.45 ± 1.01
9	12.9	Pinocarvone	1162	1160	0.25 ± 0.11	0.09 ± 0.02
10	13.1	Borneol	1168	1165	*** ND	0.11 ± 0.08
11	13.5	Terpinen-4-ol	1178	1177	1.48 ± 0.49	0.40 ± 0.01
12	14.0	*α*-Terpineol	1186	1186	0.22 ± 0.02	*** ND
13	15.3	Octanol acetate	1214	1211	0.13 ± 0.01	0.24 ± 0.01
14	17.0	Bornyl acetate	1284	1284	0.48 ± 0.16	0.18 ± 0.07
15	21.0	Longifolene	1403	1407	0.12 ± 0.08	*** ND
16	22.4	*cis*-*β*-Farnesene	1448	1454	0.58 ± 0.01	*** ND
17	23.0	Germacrene D	1480	1484	0.09 ± 0.04	*** ND

* Retention indices reported in Adams 2007. ** The concentration % were determined from the average of three replicates, and the reported results are in the form of mean ± SEM. *** ND: not detected.

**Table 2:** Docking scores of identified compounds from the essential oil of *C*. *anthemoides* leaves, stems, and flowers against COX-2 (PDB ID: 5KIR) and TNF-*α* (PDB ID: 2AZ5) receptors.

No.	Compound	COX-2 (PDB ID: 5KIR)		TNF-*α* (PDB ID: 2AZ5)	
		Pose Score (kcal/mol)	RMSD Refine (Å)	Pose Score (kcal/mol)	RMSD Refine (Å)
1	*β*-Pinene	−7.7550	0.98	−4.4942	1.44
2	*α*-Terpinene	−7.6562	1.09	−4.9016	1.29
3	D-Limonene	−7.7438	1.24	−4.9351	1.72
4	Eucalyptol	−8.4517	0.78	−4.6986	1.76
5	γ-Terpinene	−7.5690	1.03	−5.1749	1.64
6	*trans*-Thujone	−7.5807	0.71	−5.1960	2.07
7	*cis*-*para*-Menth-2-ene-1-ol	−8.7486	0.99	−6.7740	1.25
8	Camphor	−7.6977	0.89	−4.8496	1.08
9	Pinocarvone	−7.2938	0.88	−4.8235	1.77
10	Borneol	−7.8185	0.67	−4.7328	1.41
11	Terpinen-4-ol	−9.3323	0.70	−6.2579	1.18
12	*α*-Terpineol	−7.6466	0.58	−4.7338	1.48
13	Octanol acetate	−8.6976	0.84	−5.5871	0.97
14	Bornyl acetate	−9.6206	0.91	−5.1830	1.68
15	Longifolene	−9.4917	0.59	−5.8426	1.48
16	*cis*-*β*-Farnesene	−9.4392	1.09	−5.9222	1.35
17	Germacrene D	−8.9982	1.02	−5.0475	1.38
18	Resveratrol	−11.2915	0.69	−6.9306	1.17

**Table 3:** Drug-like properties of both *C. anthemoides* essential oil constituents.

No.	Name	* logP	TPSA	n atoms	MW	nHBA	nHBD	Number of Violations	nrotb	MVol
**1**	β-Pinene	3.33	0	10	136.24	0	0	0	0	152.37
**2**	*α*-Terpinene	3.36	0	10	136.24	0	0	0	1	156.74
**3**	D-Limonene	3.62	0	10	136.24	0	0	0	1	157.3
**4**	Eucalyptol	2.72	9.23	11	154.25	1	0	0	0	166.66
**5**	γ-Terpinene	3.36	0	10	136.24	0	0	0	1	156.74
**6**	*trans*-Thujone	2.16	17.07	11	152.24	1	0	0	1	160.21
**7**	*cis*-*para*-Menth-2-ene-1-ol	2.8	20.23	11	154.25	1	1	0	1	170.67
**8**	Camphor	2.16	17.07	11	152.24	1	0	0	0	159.86
**9**	Pinocarvone	2.23	17.07	11	150.22	1	0	0	0	154.55
**10**	Borneol	2.48	20.23	11	154.25	1	1	0	1	166.28
**11**	Terpinen-4-ol	2.6	20.23	11	154.25	1	1	0	1	170.65
**12**	*α*-Terpineol	2.57	0	10	136.24	0	0	0	1	157.32
**13**	Octanol acetate	3.47	26.3	13	184.28	2	0	0	7	201.74
**14**	Bornyl acetate	3.05	26.3	14	196.29	2	0	0	2	202.23
**15**	Longifolene	5.82	0	15	204.36	0	0	1	6	239.27
**16**	*cis*-*β*-Farnesene	5.84	0	15	204.36	0	0	1	7	239.82
**17**	Germacrene D	5.43	0	15	204.36	0	0	1	1	234.9

* Octanol-water partition coefficient (logP), molecular polar surface area (TBSA), number of atoms (n atoms), molecular weight (MW), number of hydrogen bond acceptors (nHBA), number of hydrogen bond donors (nHBD), number of rotatable bonds (nrotb), molecular volume (MVol).

## Data Availability

Not applicable.

## Sample Availability

Samples of the compounds are not available from the authors.
